# Morphology Control of Ni(II)-NTA-End-Functionalized Block Copolymer and Bio-Conjugation through Metal-Ligand Complex

**DOI:** 10.3390/polym9040144

**Published:** 2017-04-20

**Authors:** Dasom Park, Chaeyeon Lee, Minsu Chae, Mohammad Abdul Kadir, Ji Eun Choi, Jae Kwang Song, Hyun-jong Paik

**Affiliations:** 1 Department of Polymer Science and Engineering, Pusan National University, Busan 46241, Korea; pds@pusan.ac.kr (D.P.); chaeyeonlee@pusan.ac.kr (C.L.); minsuchae@pusan.ac.kr (M.C.); 2 Department of Chemistry, Sylhet Government Women’s College, Sylhet 3100, Bangladesh; kadir1276@gmail.com; 3 Research Center for Bio-Based Chemistry, Korea Research Institute of Chemical Technology (KRICT), Daejeon 34114, Korea; birnen@krict.re.kr (J.E.C.); ajee@krict.re.kr (J.K.S.)

**Keywords:** atom transfer radical polymerization (ATRP), end-functionalized block copolymer, nitrilotriacetic acid (NTA), self-assembly behavior, protein–polymer hybrid

## Abstract

This study demonstrates the synthesis of an amphiphilic block copolymer, Ni^2+^-nitrilotiracetic acid-end-functionalized-poly(poly(ethylene glycol)methyl ether methacrylate)-*block*-polystyrene (NTA-*p*(PEGMA-*b*-St)), morphology control via their self-assembly behavior and reversible bioconjugation of hexahistidine-tagged green fluorescent protein (His_6_-GFP) onto the surfaces of polymeric vesicles through nitrilotriacetic acid (NTA)-Ni^2+^-His interaction. First, the *t*-*boc*-protected-NTA-*p*(PEGMA-*b*-St) was synthesized by atom transfer radical polymerization. After the removal of the *t*-*boc* protecting group, the NTA group of the polymer was complexed with Ni^2+^. To induce self-assembly, water was added as a selective solvent to the solution of the copolymer in tetrahydrofuran (THF). Varying the water content of the solution resulted in various morphologies including spheres, lamellas and vesicles. Finally, polymeric vesicles decorated with green fluorescent protein (GFP) on their surfaces were prepared by the addition of His_6_-GFP into the vesicles solution. Reversibility of the binding between vesicles and His_6_-GFP was confirmed with a fluorescent microscope.

## 1. Introduction

The self-assembly of amphiphilic block copolymer has been researched intensely during the past few years as a representative method based on bottom-up approaches in the development of nanotechnology [1,2,3,4,5]. The basics of block copolymer association have something in common with the self-assembly of low molecular weight surfactants or lipids [6,7]. In comparison to small molecules, block copolymers provide some interesting morphological features such as structural stability, a broad range of morphologies and their further modification [3,7,8].

Among approaches to the development of polymeric assembly systems, structure formation using block copolymers containing special functionalities on their terminus is one of the methods used to prepare surface-functionalized nanostructures and indicates great potential of applications, depending on the functions and properties of the chain end, in diverse fields including medical, cosmetic and catalytic formulations [9,10,11,12,13,14]. In this sense, many studies have been conducted to develop functional micelles or vesicles by introducing functional groups on the terminus of the polymer. Wooley et al. reported surface-functionalized micelles from the self-assembly of block copolymer bearing 2,3-dinitrophenyl, a well-known antigenic group, or Click-reactive functional groups at the terminus [9,11]. Shoichet et al. designed bioactive immune-nanoparticles decorated with anti-HER2, a breast cancer antibody, through the self-assembly of furan functionalized block copolymer and the immobilization of antibodies on micellar surface using the Diels-Alder reaction [12]. Sumerlin et al. prepared folate-functionalized assemblies as active-targeting delivery vehicles [13]. Meier and colleagues reported functionalized polymersome from nitrilotriacetic acid (NTA)-end-functionalized poly(ethylene oxide-*block*-butadiene) diblock copolymer and its bio-conjugation with histidine (His)-tagged enhanced green fluorescent protein [14].

In the case of conventional self-assembly, amphiphilic block copolymers show diverse morphologies in the solution depending on the relative ratio of the hydrophilicity to hydrophobicity by means of several methods, such as tuning of the ratio of molecular weight of the two blocks or changing the type of solvent, solvent quality, polymer concentration, pH, ionic strength or temperature etc. [8,15,16,17,18]. Cheng et al. observed various morphologies of poly(ethylene oxide-*b*-styrene) by changing the ratio of solvent/selective solvent [19,20]. Despite the fact that the observation of morphology transition by environmental changes is meaningful, the studies have been limited to prevalent block copolymers. Thus, the application of the controlled morphology transition induced self-assembly of end-functionalized block copolymer will provide important new insight into potential research directions.

Here, we prepared a block copolymer bearing the functionality at the chain end. The self-assembly of the block copolymer in the solvent switch method provides various morphologies of surface-functionalized nanostructures. Nickel(II) complexed nitrilotriacetic acid (Ni^2+^-NTA) was selected as the end-functional group. Ni^2+^-NTA is an attractive chelator that can be complexed with ligands, especially the His tag of recombinant proteins, through coordination bonding. Moreover, conjugation based on NTA chelating chemistry exhibits several important properties such as strong (K_D_ ca. 10 × 10^−6^ M), [21] fast, [22] selective [23] and reversible [24] interaction. Due to these advantages of Ni^2+^-NTA and its mild condition driving high preservation of protein activities for association and dissociation processes, this functional group has been applied for purification [25,26,27,28,29] and detection [30,31], as well as for surface immobilization [22,32,33,34,35,36] of multi-His tagged proteins. Especially in the surface immobilization field, attaching His tagged proteins to lipid-based carriers functionalized with Ni^2+^-NTA was reported broadly as a functional and oriented immobilization system [24,32,33].

**Scheme 1:**
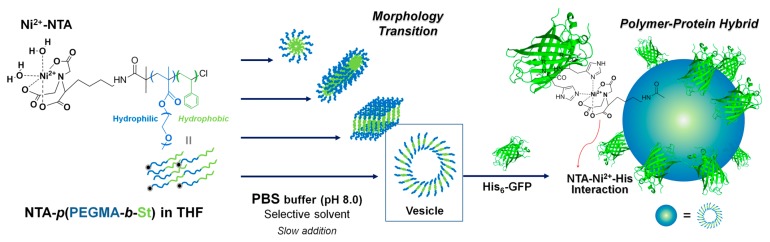
Morphology transition of Ni^2+^-nitrilotriacetic acid (NTA)-end-functionalized block copolymer and protein conjugation.

In this study, we describe the synthesis of Ni^2+^-NTA-end-functionalized-poly(poly(ethylene glycol)methyl ether methacrylate-*block*-styrene) (Ni^2+^-NTA-*p*(PEGMA-*b*-St)) by atom transfer radical polymerization (ATRP) and show that the architecture of surface-functionalized aggregates can be controlled by adjusting the solution conditon in the same tendency with common amphiphlic block copolymers such as poly(ethylene oxide-*b*-styrene). Furthermore, to confirm that the functional groups on the surface are accessible, protein–polymer hybrid nanostructures were prepared using specific interactions between immobilized Ni^2+^-NTA groups on the surface of the polymeric vesicles, as representative structures among several architectures, and hexahistidine tags located at the C-terminus of the green fluorescent protein (His_6_-GFP) (Scheme 1).

## 2. Materials and Methods

### 2.1. Materials

Poly(ethylene glycol) methacrylate with 9 EO unit (PEGMA500, *M*_n_ = 500 g/moL) were purchased from Aldrich and purified by passing twice through a column filled with basic alumina to remove the inhibitors. Styrene (Junsei, Japan; 99.5%) was purified by vacuum distillation over CaH_2_. Cu(I)Cl (Aldrich, St. Louis, MO, USA; 98%) was purified by stirring with glacial acetic acid followed by filtration and washing the resulting solids with ethanol (×3) and diethyl ether (×2). Cu(II)Cl_2_ (97%, Aldrich) was purified using a literature procedure [37,38,39]. 2,2′-dipyridyl (bpy) (>99%), *N*,*N*,*N′*,*N*″,*N*″-pentamethyldiethylenetriamine (PMDETA) (99%), *tert*-butylbromoacetate (98%), palladium (10 wt % activated carbon), 2-bromoisobutyrylbromide (98%), *N*,*N*-diisopropylethylamine (DIPEA) (99%), triethylamine (TEA) (99%), *N*-hydroxysuccinimide (98%), trifluoroacetic acid (99%) and nickel chloride hydrate (99.95%) were purchased from Aldrich (USA) and used without further purification. H-Lysine(Z)-OtBu·HCl was used as received (Bachem, Switzerland; >99%). The *t*-*boc*-NTA initiator (*N*,*N*-Bis[(*tert*-butyloxycarbonyl)methyl]-*N*′-2-bromoisobutyryl-l-lysine *tert*-Butyl Ester) for ATRP was synthesized according to the previously reported procedure (Scheme S1 and Figure S1) [35]. His_6_-GFP was expressed and purified according to the procedures reported previously (see the Supporting Information) [35].

### 2.2. Instruments

Molecular weights (*M*_n_) and molecular weight distributions (*M*_w_/*M*_n_) were determined using size exclusion chromatography (SEC, Agilent, Santa Clara, CA, USA), which was calibrated with polystyrene and poly(methyl methacrylate) standards. SEC (Agilent ChemStation for LC System) was equipped with Agilent 1100 pump, RID detector and PSS SDV (5 μm, 10^5^, 10^3^, 10^2^ Å 8.0 mm × 300.0 mm) columns. Tetrahydrofuran (THF) was used as a mobile phase at a flow rate of 1.0 mL/min and a column temperature of 313 K. Monomer conversion was determined by HP 5890 gas chromatography (Hewlett-Packard Comp., Palo Alto, CA, USA) equipped with HP101 column (methyl silicone fluid, 25 m × 0.32 mm × 0.30 μm, Agilent, Santa Clara, CA, USA). ^1^H NMR spectra were obtained on a Varian Unity Inova 500 spectrometer and a Varian Unity Plus 300 spectrometer (Varian Deutschland Gmbh, Darmstadt, Germany). ^13^C NMR spectra were obtained on a 600 MHz agilent NMR system (Agilent, Santa Clara, CA, USA). Inductively coupled plasma-mass spectrometry (ICP-MS, Element2, J200 Tandem, Applied Spectra Inc., Fremont, GA, USA) was applied for determination of nickel content in the polymer. Transmission electron microscope (TEM) and scanning electron micro (SEM) images were obtained on a Hitachi H-7600 instrument (Hitachi High-Technologies, Tokyo, Japan) at 100 kV and on a Hitachi S-4200, respectively. Dynamic light scattering (DLS) study was performed with a 90 plus Particle Size Analyzer (Brookhaven Instruments Corporation, New York, NY, USA). Fluorescence optical microscope images were obtained on a Leica system (DM5500B/DFC490/CW4000, Leica Microsystems, Wetzlar, Germany). The range of excitation filter wavelengths was 440–520 nm and the range of emission filter wavelengths was 497–557 nm. The photoluminescence (PL) spectra were obtained with an excitation wavlength of 470 nm (Ocean Optics HR4000CG Composite-grating spectrophotometer, Ocean Optics Inc., Dunedin, FL, USA).

### 2.3. Synthesis of t-boc-NTA-End-Functionalized p(PEGMA)

**Scheme 2:**
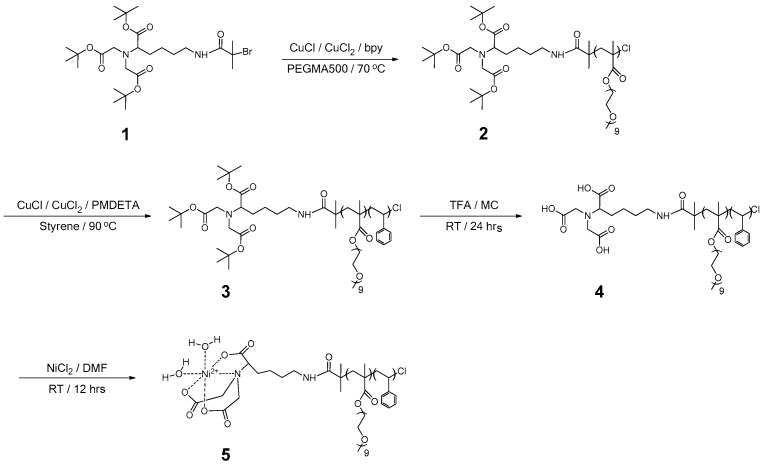
Synthesis of Ni^2+^-nitrilotiracetic acid-end-functionalized-poly(poly(ethylene glycol)methyl ether methacrylate)-*block*-polystyrene (NTA-*p*(PEGMA-*b*-St)).

The synthesis of *t*-*boc*-NTA-end-functionalized *p*(PEGMA) (macroinitiator) (**2**) is shown in Scheme 2. (**1**) (220 mg, 0.380 mmoL), PEGMA500 (24.6 mL, 53.1 mmoL), anisole (8.8 mL) and bpy (122.5 mg, 0.774 mmoL) were added to a N_2_-purged Schlenk flask. After three freeze-pump-thaw cycles, CuCl (37.6 mg, 0.380 mmoL) and CuCl_2_ (1 mg, 0.0076 mmoL) were added to the flask. Then, when three freeze-pump-thaw cycles were done, the flask was placed in to an oil bath set at 70 °C and stirred for 6.5 h. The mixture was diluted with THF and passed through a column of neutral alumina to remove Cu salt. The resulting macroinitiator was precipitated against isopropyl ether and dried in vacuo at 30 °C for 24 h.

### 2.4. Synthesis of t-boc-NTA-p(PEGMA-b-St) Block Copolymer (**3**)

The synthesis of *t*-*boc*-NTA-*p*(PEGMA-*b*-St) block copolymer (**3**) is shown in Scheme 2. (**2**) (200 mg, 0.01 mmoL), styrene (3.87 mL, 33.8 mmoL), anisole (4 mL) and PMDETA (13.84 mg, 0.08 mmoL) were added to a N_2_-purged Schelnk flask. After three freeze-pump-thaw cycles, CuCl (7.3 mg, 0.0736 mmoL) and CuCl_2_ (1 mg, 0.0074 mmoL) were added to the flask. It was followed by three more freeze-pump-thaw cycles and then placed into an oil bath set at 90 °C and stirred for 24 h. The mixture was diluted with THF and passed through a column of neutral alumina to remove Cu salt. The product was purified via precipitation against methanol and dried in vacuo at 30 °C for 24 h.

### 2.5. Synthesis of Ni^2+^-NTA-p(PEGMA-b-St) (**5**)

The removal of protecting groups of NTA moieties was performed as reported in the literature [35]. *t*-*boc*-NTA-*p*(PEGMA-*b*-St) (**3**) (200 mg, 1.3 × 10^−^^3^ mmoL) and trifluoroacetic acid (TFA, 40 µL) were stirred in 10.0 mL of CH_2_Cl_2_ in oil bath set at 30 °C. The product was precipitated against MeOH and dried in vacuo at 30 °C for 24 h. Finally Ni^2+^-NTA-*p*(PEGMA-*b*-St) (**5**) was prepared by the addition of nickel chloride (NiCl_2_, 1 mg, 7.7 × 10^−3^ mmoL) to the solution of NTA-*p*(PEGMA-*b*-St) (**4**) (100 mg, 0.66 × 10^−^^3^ mmoL) in DMF in a glass vial. The product was precipitated against MeOH and dried in vacuo at 30 °C for 24 h.

### 2.6. Self-Assembly of Block Copolymer

0.5 mg of Ni^2+^-NTA-*p*(PEGMA-*b*-St) (**5**) and 4.5 mg of *t*-*boc*-NTA-*p*(PEGMA-*b*-St) (**3**) were first dissolved in THF (5 mL) to obtain 0.1 wt % of copolymer concentration and stirred at room temperature. To obtain various morphologies, Phosphate buffer solution (50 mM PBS, pH 8.0) was added slowly (0.5 mL/h) to the copolymer solution using a syringe pump [19]. A small amount of sample was taken at a predetermined water concentration, after equilibration time of at least 30 min for formation of particular nanostructures, and excess PBS (100 times to the sample) was quickly added to quench the formed morphology [18]. Buffer solution and THF were filtrated through a 0.2 μm pore size syringe filter before the experiment.

### 2.7. Bioconjugation of Polymeric Vesicles with His_6_-GFP 

The THF was removed from the quenched polymeric vesicles solution using a rotary evaporator in vacuo at room temperature for 1 h. 2.5 μL of PBS (10 mM, pH 7.5) containing His_6_-GFP (1.8 mg/mL) was subsequently added to 2.5 mL of polymeric vesicles solution and stirred with 250 rpm at room temperature for 24 h. The structural stability of protein–polymer hybrid vesicle was estimated through the stability of GFP fluorescence measured by PL spectroscopy.

### 2.8. Release of His_6_-GFP from the Hybrid

3 mg of imidazole was added to 2.5 mL of His_6_-GFP conjugated vesicle solution and stirred at room temperature for 1 day. The samples were examined using fluorescence microscope.

## 3. Results and Discussion

### 3.1. Synthesis Nitrilotiracetic Acid (NTA)-Functionalized Block Copolymer

ATRP is a useful method to obtain block copolymers since most of the chain after polymerization contains halide end-functionality which can be introduced as a macroinitiator. The introduction of end-functionality at α-position of the polymer chain can also be achieved easily through functionalities of the alkyl side of the ATRP initiator [40]. The amidic initiator containing alkyl bromide was synthesized to introduce a NTA functional group at polymer chain end [35]. The initiator was prepared with *t*-*boc* protecting groups in order to ensure high solubility of the initiator and prevent protonation of the ligand in the ATRP step (See Scheme S1) [41,42]. Scheme 2 shows the overall synthesis steps of Ni^2+^-NTA-*p*(PEMGA-*b*-St) (**5**). First, the hydrophilic *p*(PEGMA) block was synthesized using (**1**) as the initiator in an ATRP reaction. In the ATRP reaction, halogen exchange technique was employed using CuCl/CuCl_2_/bpy to overcome slow initiation. As a result, the Br group which initially provides fast initiation is replaced predominantly with more stable Cl as the end group in the growing polymer chains. So initiation efficiency is improved as the rate of initiation is increased relative to the propagation rate [43,44]. The chain extension of PEGMA with styrene block was carried out using CuCl/CuCl_2_/PMDETA and *t*-*boc*-NTA-*p*(PEGMA)-Cl (**2**) as macroinitiator.

**Figure 1:**
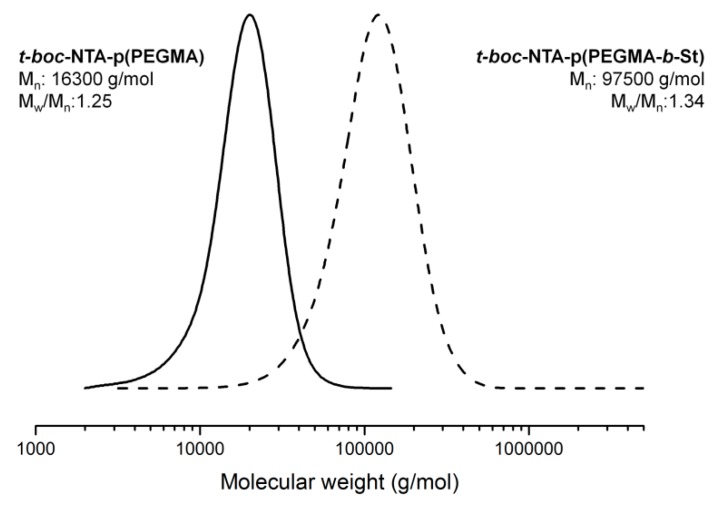
Size exclusion chromatography (SEC) traces of polymers. Solid line for *t*-*boc*-NTA-*p*(PEGMA) (**2**); dash line for *t*-*boc*-NTA-*p*(PEGMA-*b*-St) (**3**).

The SEC traces of macroinitiator and block copolymer are shown in Figure 1. The shift of SEC trace to higher molecular weight confirmed the successful polymerization of the styrene block with the macroinitiator. The number average molecular weights (*M*_n_) of the macroinitiator (**2**) and block copolymer (**3**) were determined as 16,300 g/moL and 97,500 g/moL, whereas molecular weight distributions (*M*_w_/*M*_n_) were 1.25 and 1.34, respectively.

**Figure 2:**
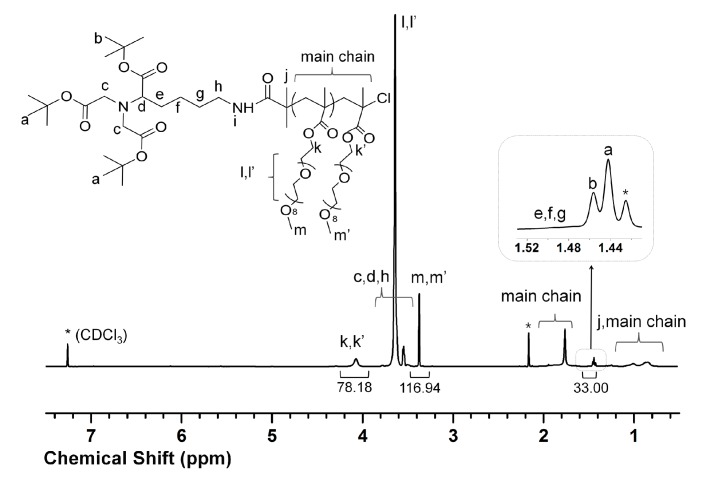
^1^H NMR (500 MHz) spectrum of *t*-*boc*-NTA-*p*(PEGMA) (**2**) in, CDCl_3_.

Distinctive peaks verified the presence of NTA moiety in *t*-*boc*-NTA-*p*(PEGMA) (**2**) in Figure 2. Peaks at 1.460 ppm (a) and 1.446 ppm (b) corresponding to *t*-butyl protons in the ^1^H NMR spectrum indicated that *t*-*boc*-NTA initiator was successfully used as an ATRP initiator. The existence of NTA functionality at the macroinitiator was also confirmed through ^13^C NMR analysis (Figure S2). The degree of polymerization (DP) and number average molecular weight (*M*_n_) of macroinitiator (**2**) were calculated by using the integral ratio of a, b, e, f, g peaks (1.52–1.442 ppm) to the methylene peaks at 4.071 ppm (k, k’, –CH_2_–) in *p*(PEGMA) (DP = 39, *M*_n*,*NMR_ = 20,000 g/moL). The *M*_n*,*NMR_ was in slight difference with *M*_n,SEC_ (=16,300 g/moL) because the standard material used for calibration of SEC was not *p*(PEGMA).

**Figure 3:**
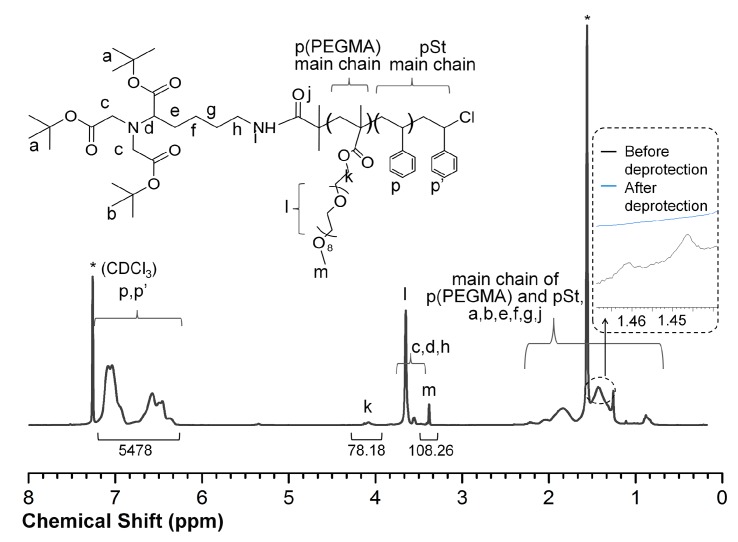
^1^H NMR (500 MHz) spectrum of *t*-*boc*-NTA-*p*(PEGMA-*b*-St) (**3**) and NTA-*p*(PEGMA-*b*-St) (**4**) in CDCl_3_.

The degree of polymerization (DP) of polystyrene block and *M*_n_,_NMR_ of (**3**) were calculated as 1096 and 134,000 g/moL by using the integral ratio of peaks at 4.081 ppm (k, k’, –CH_2_–) in *p*(PEGMA) to the phenyl ring proton (6.25–7.40 ppm) (Figure 3). The chain extension of PEGMA with styrene block was also confirmed through ^13^C NMR analysis (Figure S3). Removal of *t*-butyl protons in (**4**) was confirmed by the disappearance of peaks at 1.460 ppm (a) and 1.446 ppm (b) in the ^1^H NMR spectrum (Figure 3). For chelation of NTA groups in the block copolymer, NiCl_2_ was added to the solution of NTA-*p*(PEGMA-*b*-St) dissolved in DMF. The addition of the NiCl_2_ caused the immediate color change of the solution from pale yellow to green [14]. The 321 ppm of nickel was included in the polymer and it means that nickel was complexed with 82.7% of the polymer chain end.

### 3.2. Morphology Transition of Block Copolymer via Self-Assembly in THF/Water

To obtain various nanostructures based on the block polymer, we prepared a 0.1 wt % solution of the polymer mixture containing 90 mol % of *t*-*boc*-NTA-*p*(PEGMA-*b*-St) and 10 mol % of Ni^2+^-NTA-*p*(PEGMA-*b*-St) in THF in a sealed vial. The PBS solution was subsequently added to the polymer solution under continuous stirring using a syringe pump. When a predetermined concentration of water was reached, the solution was stirred for 30 min to equilibrate. To observe micelle morphologies retaining original shapes and sizes, a small amount of each sample was quenched after a particular interval with excess PBS for quick vitrification of *p*St block [45].

Both *p*(PEGMA) block and *p*St block are very soluble in THF; self-assembly does not occur with a high content of THF. When water, a poor solvent for *p*St block, is added continuously up to the critical water concentration (CWC), an onset of self-assembly occurs due to their unique amphiphilic features. After the micelles started to form at the CWC, further increase of water content in the system resulted in gradual change in the shape of the micelles from sphere to cylinder, to lamella and to vesicle [18].

**Figure 4:**
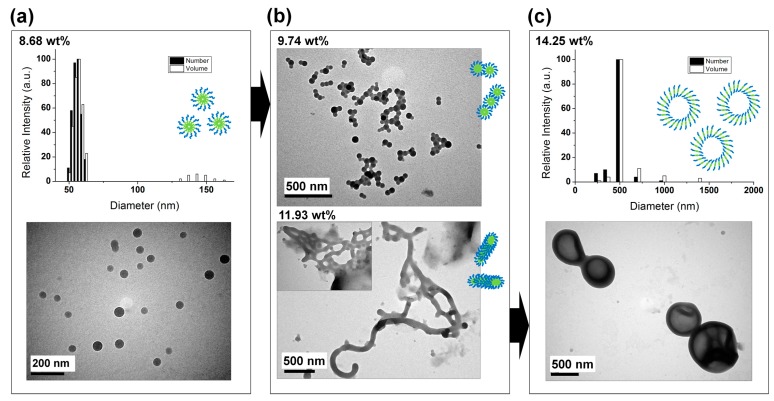
Morphology observation through dynamic light scattering (DLS) and transmission electron microscope (TEM) measurement of 0.1 wt % of *t*-*boc*-NTA-*p*(PEGMA-*b*-St)/Ni^2+^-NTA-*p*(PEGMA-*b*-St) according to water concentration; (**a**) spherical micelles at 8.68 wt % water; (**b**) morphology transition at from 9.74 wt % to 11.93 wt % water morphology transition for 9.74–11.93 wt % water; (**c**) vesicles at 14.25 wt % water.

At relatively low concentrations of water (8.68 wt %), spherical micelles with diameter of 50~62 nm were observed by DLS and TEM (Figure 4a). After the CWC point, the morphology transition according to the increase in water content was observed in the TEM analysis of each quenched sample (Figure 4b). When the water concentration was increased to 9.74 wt %, spherical micelles started to aggregate and at a water concentration of 11.93 wt %, the aggregate changed into a shape with a cylindrical structure. In this stage, the transition of cylinder to bilayer morphology was observed. The phenomenon of transformation into more macroscopic structures was well-matched with the self-assembly behavior of common block copolymers. When the water concentration reached 14.25 wt %, the DLS and TEM analysis showed that the morphology became dominant with vesicles of ca. 500 nm size (Figure 4c). We could observe that the solution became abruptly more turbid. The DLS autocorrelation functions of micelles and vesicles were shown at Figure S4. A more detailed investigation through SEM measurement of the vesicle’s morphology obtained at a water content of 14.25 wt % is shown in Figure S5.

This phenomenon can be explained using the free energy of micelles. The free energy can be considered by dividing it into three components—the free energy of core forming chains, the interfacial energy between core and solvent, and the energy of corona forming chains. Only the energy between core and solvent will be considered because it is the dominant component when compared to the others [19]. As water content increases in the block copolymer/THF solution, the overall solvent system becomes poorer for the *p*St block [46]. It induces increase in the interfacial free energy between solvent and *p*St block which compose the core of micelles, and as the size of the micelle increases, the degree of stretching of the *p*St chains increases [47]. In other words, the morphology of micelles changes from spherical micelles with a small diameter to other morphologies with bigger dimensions, due to the tendency to reduce high interfacial energy caused by the addition of poor solvent.

### 3.3. Bioconjugation with His_6_-GFP

It is important to examine whether surface functionalized-nanostructures can realize their functions to be applied in proper fields such as protein immobilization, nano-reactors and drug delivery systems. In this sense, we tried to conjugate His_6_-GFP on the surface of Ni^2+^-NTA functionalized vesicles as a representative structure for easy demonstration, and investigated their specific and reversible conjugation via fluorescence microscope analysis.

**Figure 5:**
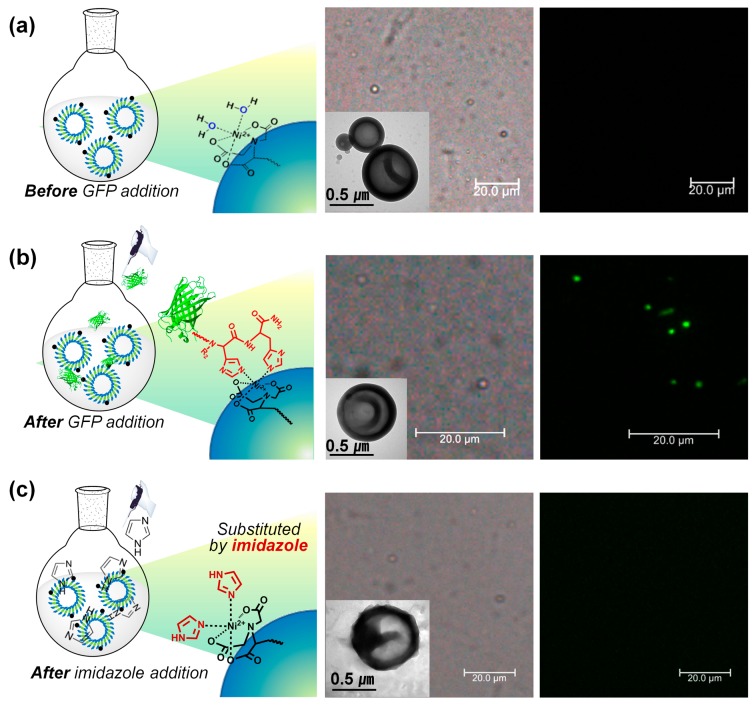
Preparation of bio conjugate between hexahistidine-tagged green fluorescent protein (His_6_-GFP) and polymeric vesicles and release of His_6_-GFP from the GFP-polymeric vesicles conjugate. TEM, optical and fluorescence microscope images of before His_6_-GFP addition (**a**), after His_6_-GFP addition to vesicle solution (**b**) and after imidazole addition to GFP-vesicle solution (**c**).

THF in the quenched polymeric vesicle solution in excess water (formed at 14.25 wt % of water) was removed using rotary evaporator at room temperature. Preservation of the shape and diameter of vesicles after evaporation was confirmed by TEM images (Figure 5a). The His_6_-GFP solution was then added to the vesicle solution and the solution was stirred for 24 h to supply enough time to conjugate GFP on the surface of vesicle. Conjugation between His_6_-GFP and polymeric vesicles was confirmed by fluorescence microscopy (Figure 5). Green fluorescence was not observed before the addition of His_6_-GFP into the vesicle solution (Figure 5a), but after the addition of His_6_-GFP, green-fluorescent dots with same diameter of the vesicles appeared (Figure 5b). The GFP fluorescence of hybrid vesicles was maintained until 4 days after conjugation but rapidly decreased after 1 week, while the fluorescence of free-GFP was well maintained (Figure S6).

Although these results meant the vesicles produced indicated a good immobilization ability of His_6_-GFP on their surfaces, it was not clear that the conjugation occurred by specific NTA-Ni^2+^-His interaction. To demonstrate that conjugates were formed by specific interaction between Ni^2+^-NTA and 6 × histidine tags, His_6_-GFP dissociation from vesicles was performed through ligand substitution reaction. Excess imidazole was added into the His_6_-GFP/polymeric vesicle conjugate solution as a competitive ligand and stirred for 1 day to supply enough time for the complete exchange of ligand. The fluorescence intensity of the conjugates vanished (Figure 5c) while retaining the original shape and size (Figure 5c). These results could be proof that the binding between His_6_-GFP and polymeric vesicles were formed *via* the specific NTA-Ni^2+^-His interaction by preserving Ni^2+^-NTA function of the polymer chain end.

## 4. Conclusions

We synthesized Ni^2+^-NTA-end-functionalized amphiphilic block copolymer from an ATRP reaction with initiators having protected NTA moieties, followed by the removal of protecting groups of the NTA moieties and the complexation of nickel ion, and characterized by SEC, ^1^H and ^13^C NMR spectroscopy. To induce self-assembly, the block copolymer was first dissolved in THF and then water was added slowly into the solution. By adjusting the water concentration in the self-assembly process, we observed morphology transition by the formation of various structures of NTA-end-functionalized block copolymer well-matched with the self-assembly behavior of common block copolymers. To confirm the surface functionality of the prepared structures, His_6_-GFP was added into the vesicle solution to induce NTA-Ni^2+^-His interaction, and successful conjugation was verified by fluorescence microscopy. This study proved the facile approach to control the shape and size of surface-functionalized structures by introducing the self-assembly behavior of end-functionalized amphiphilic block copolymer. These diverse structures can be valuable for proper applications, depending on their end-functionality and morphology. NTA functionalized structures indicate especially high potential in protein immobilization, nano-reactors and drug delivery systems, through the specific interaction between polymer and protein.

## Supplementary Materials

The following are available online at www.mdpi.com/2073-4360/9/4/144/s1, Scheme S1: Synthesis of *t*-*boc*-nitrilotriacetic acid (NTA) initiator for ATRP, Figure S1: ^1^H NMR (300 MHz) spectrum of *t*-*boc*-NTA (**1**) initiator in CDCl_3_, Figure S2: ^13^C NMR (150 MHz) spectrum of *t*-*boc*-NTA-*p*(PEGMA) (**2**) in CDCl_3_, Figure S3: ^13^C NMR (150 MHz) spectrum of *t*-*boc*-NTA-*p*(PEGMA-*b*-St) (3) in CDCl_3_, Figure S4: The DLS autocorrelation functions of micelles (at 8.68 wt % water) and vesicles (at 14.25 wt % water), Figure S5: SEM images of vesicles formed at 14.25 wt % water under different magnification, Figure S6: Fluorescence stability of GFP-vesicles and free GFP as time passed.
