# Supplementary material for: Morphology Control of Ni(II)-NTA-End-Functionalized Block Copolymer and Bio-Conjugation through Metal-Ligand Complex
Source: Polymers (Basel). 2017 Apr 20;9(4):144. doi: 10.3390/polym9040144 (PMC6432091; doi:10.3390/polym9040144)
Supplement: Supplementary file 1 [file polymers-09-00144-s001.pdf]

Supplementary Materials: Morphology control of Ni(II)-NTA-end-functionalized block copolymer and bio-conjugation through metal-ligand complex

Dasom Park, Chaeyeon Lee (co-first author), Minsu Chae, Mohammad Abdul Kadir, Ji Eun Choi, Jae Kwang Song and Hyun-jong Paik

Experimental

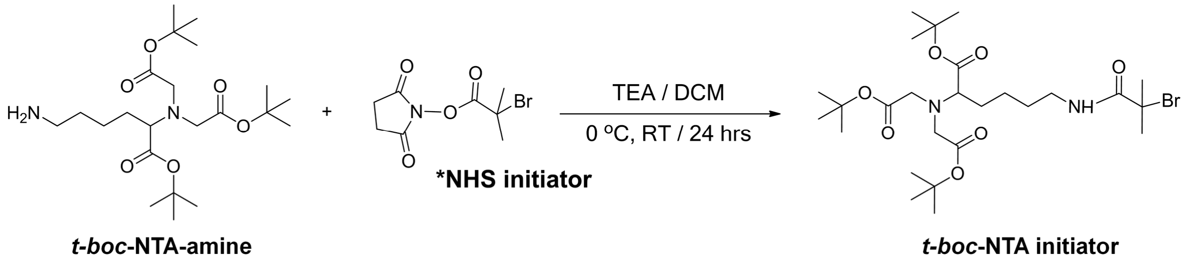

Scheme 1. Synthesis of *t-boc*-nitrilotriacetic acid (NTA) initiator for ATRP.

Synthesis of *t-boc*-NTA initiator for ATRP

t-boc-NTA initiator for ATRP was prepared by following our previous reported procedure.[1] *t-boc*-NTA-amine[2] and NHS initiator[2] were first synthesized by following the reported procedure. Mixture of *t-boc*-NTA-amine (950 mg, 2.2721 mmol) and triethylamine (TEA) (317 μL, 2.2721 mmol) in 5.0 mL dichloromethane (DCM) was added drop wise to a stirring NHS-initiator (0.5 g, 1.9 mmol) dissolved in 100 mL DCM in an ice bath for 1 h. Upon complete addition of the solution, the mixture was stirred at room temperature for 24 h. The mixture was washed with water (5 × 100 mL) and the crude product was purified by column chromatography by eluting with 3:1 n-hexane/ethyl acetate. ^{1}H NMR (300 MHz, $CDCl_3$), δ (ppm): 1.38 (s, 18H), 1.40 (s, 9H), 1.49 (m, 2H), 1.60 (m, 2H), 1.65 (m, 2H), 1.88 (s, 3H) 3.20 (t, 2H), 3.25 (t, 1H), 3.42 (dd, 4H), 4.41 (q, 1H).

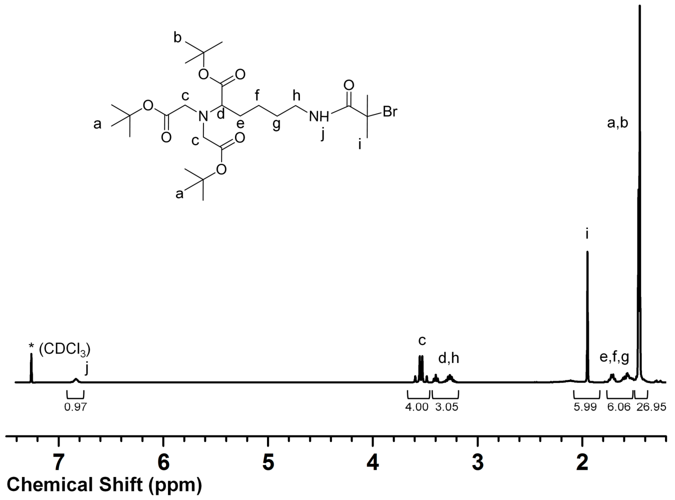

Figure S1. ^{1}H NMR (300 MHz) spectrum of *t-boc*-NTA (1) initiator in $CDCl_3$.

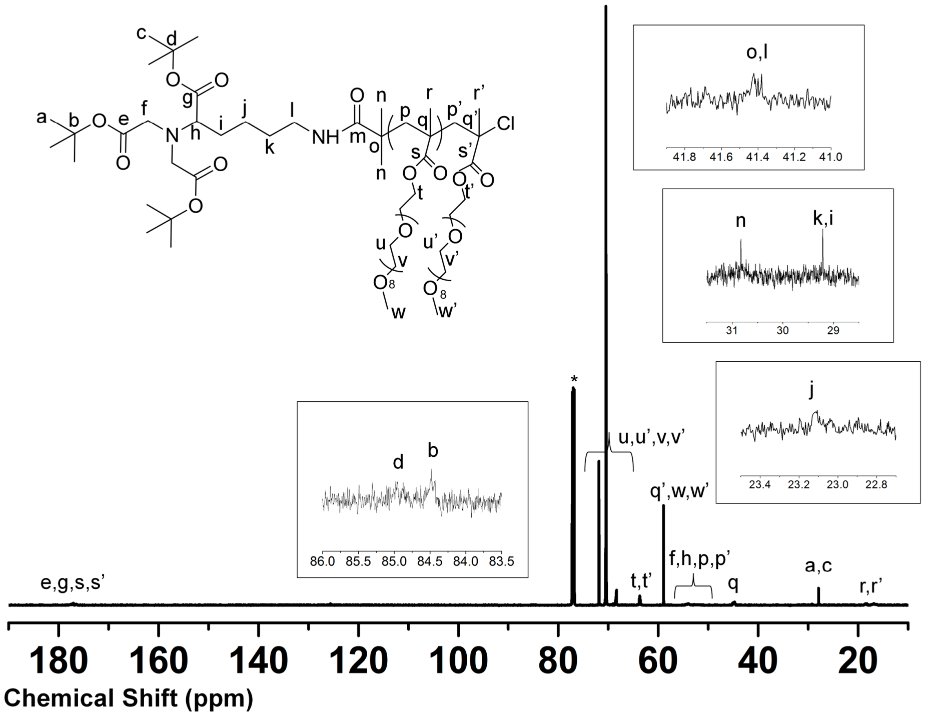

Figure S2. ^{13}C NMR (150 MHz) spectrum of *t-boc*-NTA-*p*(PEGMA) (2) in $CDCl_3$.

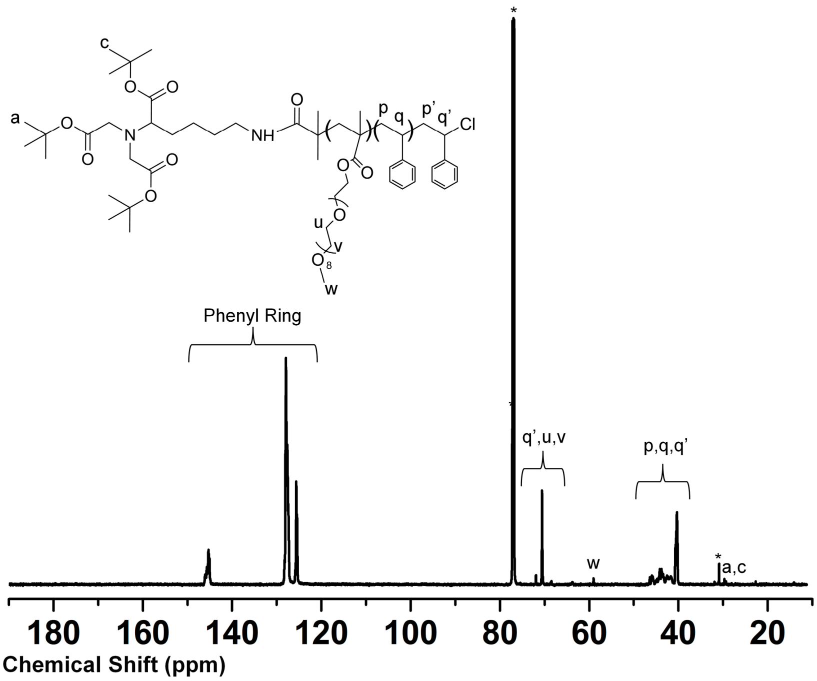

Figure S3. ^{13}C NMR (150 MHz) spectrum of *t-boc*-NTA-*p*(PEGMA-*b*-St) (3) in $CDCl_3$.

Expression and Purification of His$_6$-GFP

Escherichia coli BL21 (DE3) harboring the pET-GFPmut3.1 expressing GFPmut3.1 tagged with 6 x histidine at its C-terminus was grown to OD600 of 0.6~0.8 in 500 mL of Luria-Bertani (LB)

medium containing 50 g/mL of canamycin, induced with 1 M isopropyl-D-thiogalactopyranoside (IPTG) at 37 °C for overnight and pelleted using centrifuge (7,000 g for 30 min). Collected cell pellet was rinsed with diionized water and centrifuged (7,000 g for 30 min). After that, the pellet was resuspended in 20 mL of Tris-HCl buffer solution (pH 8.0, 20 mM) with sonication and centrifuged again (15,000 g for 20 min). After the extracts was incubated with 5 mL of Ni-NTA His-bind resin, the resin was loaded into column, washed with 250 mL of Lysis buffer (50 mM phosphate buffer, pH 8.0; 300mM NaCl; 10 mM imidazole), 250 mL of washing buffer (50 mM phosphate buffer, pH 8.0; 300 mM NaCl; 20 mM imidazole), and the His_6-GFP was eluted with 6 × 5 mL of elution buffer (50 mM phosphate buffer, pH 8.0; 300 mM NaCl; 250 mM imidazole). Imidazole in the eluted solution was removed by Slide-A-Lyzer Dialysis Cassette (MWCO 10,000) and concentrated using centrifuge. The protein fractions were analyzed by SDS-PAGE.

Preparation of TEM and SEM Samples

TEM and SEM samples were prepared by dropping solutions onto the TEM carbon coated gird. Extra solution was blotted with filter paper and the grids were subsequently dried for 24 h at room temperature. The samples were without staining.

DLS Measurement

DLS experiments were carried out using a home-built setup fitted with a laser operating at 660 nm. Samples were loaded into a spherical glass cuvette before measurement without dilution. All measurements were performed at angles of 90° at 25 °C. Each measurement was the sum of 5 runs, and the duration of single run was one min. The size of polymeric vesicle was measured in terms of number and volume distribution. Figure S4 indicates the DLS autocorrelation functions of micelles and vesicles.

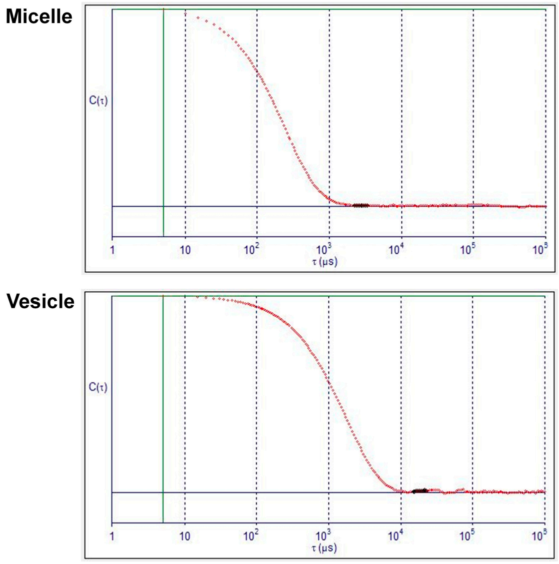

Figure S4. The DLS autocorrelation functions of micelles (at 8.68 wt% water) and vesicles (at 14.25 wt% water)

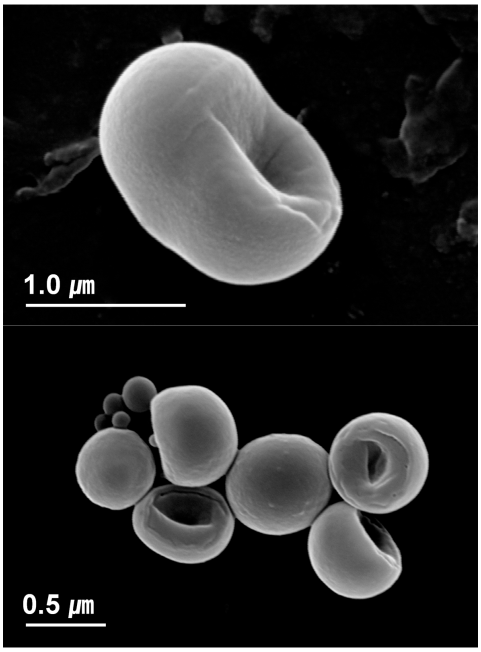

Figure S5. SEM images of vesicles formed at 14.25 wt% water under different magnification.

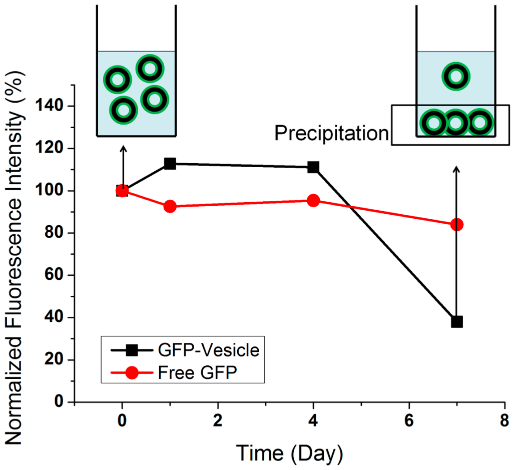

Figure S6. Fluorescence stability of GFP-vesicles and free GFP as time passed.

Reference

1. Cho, H.Y.; Kadir, M.A.; Kim, B.-S.; Han, H.S.; Nagasundarapandian, S.; Kim, Y.-R.; Ko, S.B.; Lee, S.-G.; Paik, H.-j. Synthesis of well-defined (nitrilotriacetic acid)-end-functionalized polystyrenes and their bioconjugation with histidine-tagged green fluorescent proteins. *Macromolecules* **2011**, *44*, 4672-4680.
2. Luk, Y.-Y.; Tingey, M.L.; Hall, D.J.; Israel, B.A.; Murphy, C.J.; Bertics, P.J.; Abbott, N.L. Using liquid crystals to amplify protein-receptor interactions: Design of surfaces with nanometer-scale topography that present histidine-tagged protein receptors. *Langmuir* **2003**, *19*, 1671-1680.
